# Non-Embedded Silver Nanowires/Antimony-Doped Tin Oxide/Polyethylenimine Transparent Electrode for Non-Fullerene Acceptor ITO-Free Inverted Organic Photovoltaics

**DOI:** 10.1021/acsaelm.2c01187

**Published:** 2023-01-11

**Authors:** Efthymios Georgiou, Apostolos Ioakeimidis, Ioanna Antoniou, Ioannis T. Papadas, Alina Hauser, Michael Rossier, Flavio Linardi, Stelios A. Choulis

**Affiliations:** † Molecular Electronics and Photonics Research Unit, Department of Mechanical Engineering and Materials Science and Engineering, Cyprus University of Technology, 45 Kitiou Kyprianou Street, Limassol 3603, Cyprus; ‡ Department of Public and Community Health, School of Public Health, University of West Attica, Athens 11521, Greece; § Avantama AG, Laubisruetistr. 50, Staefa 8712, Switzerland

**Keywords:** ITO-free organic photovoltaics, silver nanowires, doped metal oxides, carrier-selective contact, electrodes, inverted organic solar cells, printed electronics

## Abstract

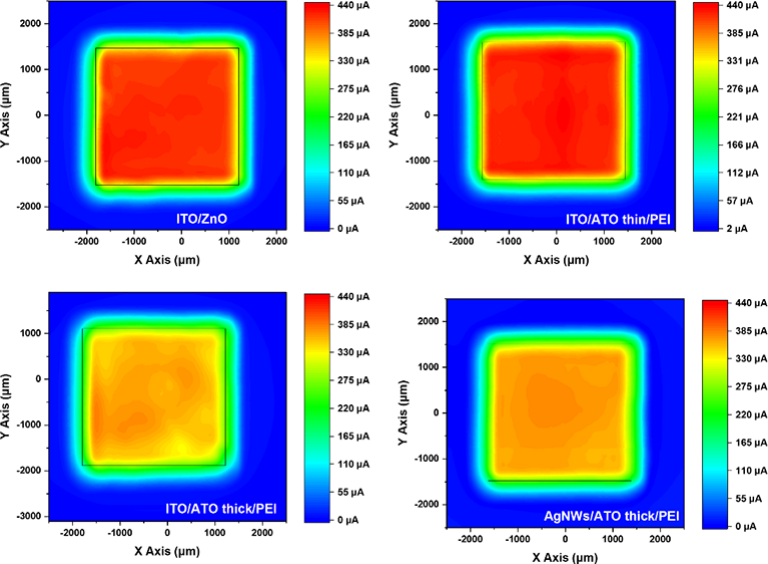

Indium tin oxide (ITO)-free solution-processed transparent electrodes are an essential component for the low-cost fabrication of organic optoelectronic devices. High-performance silver nanowires (AgNWs) ITO-free inverted organic photovoltaics (OPVs) usually require a AgNWs-embedded process. A simple cost-effective roll-to-roll production process of inverted ITO-free OPVs with AgNWs as a bottom transparent electrode requires solution-based thick metal oxides as carrier-selective contacts. In this reported study, we show that a solution-processed antimony-doped tin oxide (ATO)/polyethylenimine (PEI) electron-selective contact incorporated on the top of non-embedded AgNWs provides a high-performance ITO-free bottom electrode for non-fullerene acceptor (NFA) inverted OPVs.

## 1 Introduction

Encouraging progress on the development of small-molecule non-fullerene acceptors (NFAs) during the past years has boosted the power conversion efficiency (PCE) to 19% of the solution-processed bulk heterojunction organic photovoltaics (OPVs).^1−3^ The high performance of the non-fullerene acceptor OPVs is of great significance for industrial applications and introduces new commercialization opportunities for OPVs. The commercialization feasibility of OPVs requires low-cost and roll-to-roll compatible materials. An important research and development milestone toward up-scalability and roll-to-roll manufacturing of OPVs is the avoidance of energy-consuming deposition techniques and of high-cost electronic materials. The development of cost-effective solution-processed electrodes is one of the key OPV product development targets providing the potential for future low-cost next-generation photovoltaics.^4,5^

A transparent conductive electrode is an essential component of optoelectronic devices. Indium tin oxide (ITO) is the most widely used transparent conductor in various optoelectronic applications with a transparency >80% and sheet resistance values ranging from 4 to 30 Ω/sq. However, its high cost and brittleness render it incompatible for the development of cheap, printable, and flexible electronic applications. Therefore, solution processability is a key characteristic for successful alternative TCOs. Among them, metal nanowires and printed metal nanoparticles are two of the most promising alternatives as reported in the literature.^6−13^

Nanowire- and microwire-based inorganic material (e.g., oxides and selenides) structures have been used for various applications such as photodetectors and photoferroelectric solar cells.^14−19^ Metal nanowires, and especially silver nanowires (AgNWs), are a suitable replacement of ITO due to their fascinating optical and electrical properties.^20^ High-efficiency normally structured OPVs utilizing AgNWs as a bottom transparent electrode have already been successfully demonstrated. In these devices, the AgNWs are coated by PEDOT:PSS, forming the hole-selective contact, while its versatile processability allows the efficient coverage of AgNWs and surface smoothening.^21^

Incorporating AgNWs in inverted structured OPVs yields processing limitations and detrimental effects on the device. The thin (20–40 nm) solution-processed metal oxides, which are commonly used as electron-selective contacts such as ZnO, cannot efficiently cover AgNWs, leading to shunt OPV devices.^22^ A possible strategy to encounter the above-mentioned limitation is by embedding the AgNWs in a transparent polymer such as polyimide.^23,24^ However, the embedding process complicates the fabrication process of OPVs and is a limiting factor for roll-to-roll manufacturing of OPVs.

Another approach to encountering the AgNW surface roughness could be the use of thick electron-selective contacts. However, conventionally thick wide band-gap metal-oxide buffer layers limit OPV performance due to their low electrical conductivity. Therefore, highly conductive metal-oxide electron-selective contacts are essential for high-performance AgNWs-based inverted OPVs. A common strategy to increase the conductivity of conventional metal oxides is by suitable doping. Doping of tin oxide (SnO_2_) with 10% antimony (Sb) increases the electrical conductivity of metal-oxide ATO (Sb:SnO_2_) up to 10^–3^ S/cm, a relatively high value for a wide band-gap metal-oxide semiconductor.^25^ In a previous report, we have demonstrated the use of antimony-doped tin oxide/polyethylenimine (ATO/PEI) as a high-performance light soaking-free electron-selective contact for inverted P3HT:PCBM and P3HR:IDTBR ITO-based OPVs.^26^

In this work, we present the fabrication of a front transparent electrode in a structure consisting of AgNWs/ATO/PEI with the scope to fabricate efficient ITO-free inverted OPVs with a thick (∼130 nm) electron-selective contact (ESC) considering that thick layers are a prerequisite for large-area OPV production. The deposition of AgNWs/ATO/PEI as well as the active layer was performed in air (ambient conditions), while the back electrode formed by thermally evaporated MoO_3_/Ag. Initially, the AgNW processing optimization study was conducted using the standard reference normal OPV AgNWs/PEDOT:PSS/P3HT:PCBM/Ca/Al, and then the optimized AgNW deposition parameters were used for fabrication of the inverted OPVs AgNWs/ATO/PEI/PM6:Y6/MoO_3_/Ag. The investigation was performed by comparing the OPV devices incorporating thin (∼40 nm) or thick (∼130 nm) ATO/PEI deposited on top of spin-coated AgNWs or commercially available sputtered ITO. The AgNWs-based inverted ITO-free OPVs with a PM6:Y6 active layer incorporating thick ATO/PEI electron-selective contacts provided a PCE of 10.23%, with the corresponding device incorporating ITO/thick ATO/PEI delivering 11.27%, which is only ∼10% higher compared to the former. Moreover, the thin ATO/PEI-based ITO-free inverted OPVs provided very limited functionality due to shunted devices, further confirming the need for implementation of thick selective contacts on top of solution-processed AgNWs for large-area OPVs.

## 2 Results and Discussion

### 2.1 Ag Nanowire Transparent Electrode Properties and Processing Optimization

**Figure 1:**
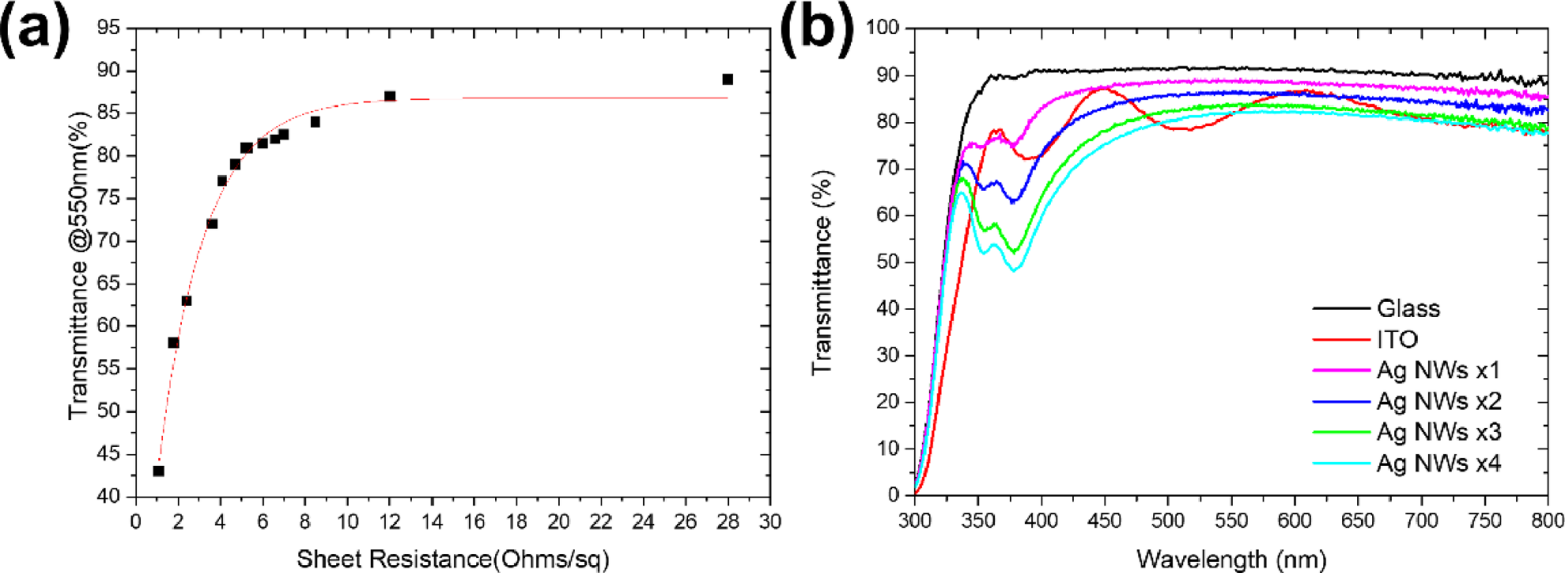
(a) Optical transmittance (λ = 550 nm) versus sheet resistance for AgNW conducting transparent electrodes. (b) Optical transmittance spectrum of glass, ITO, and AgNWs deposited one, two, three, and four times.

Optimization of AgNW processing parameters is necessary to define the ideal balance between the optical transparency and the electrical conductivity of AgNWs-based electrodes. A higher AgNW density provides higher electrical conductivity but lower transparency. Figure 1a describes the relationship between the optical transmittance (*T*) and the sheet resistance *R*_sq_ for the AgNW films. The transmittance value of 550 nm wavelength was chosen for the *y*-axis of the graph since 550 nm is an indicated maximum absorbance value for organic semiconductors in the visible spectrum. In addition, the transmittance of AgNWs is relatively stable in the range of 400–800 nm as shown in Figure 1b. Low sheet resistance and high transmittance values are desired for efficient optoelectronic applications. Figure 1a shows the transmittance at 550 nm versus the sheet resistance of various nominal thicknesses of AgNW layers fabricated by changing the spin speed and the number of sequential depositions. Our findings on the relation between the transmittance and the sheet resistance are in good agreement with Cambrios’ AgNW provided internal data and the previously published literature on AgNWs.^27,28^Figure 1b indicates high transparency in the visible spectrum for AgNW electrodes with various thicknesses (one, two, three, and four times) in comparison with the reference ITO transparent electrode and glass substrate. The corresponding sheet resistance values of the prepared AgNW film are presented in Table S1. As illustrated in Figure 1b, comparing the transmittances (at 550 nm) of AgNW electrodes with the glass substrate, the transmittance loss due to AgNWs ranges between 2 and 8%. The measurements performed in air and, thus, part of the transmission loss is due to reflection, since according to Cambrios, at 10–20 ohm/sq, the actual transmission loss due to AgNWs is only around 2% (Karl Pichler of Cambrios private communications).

Moreover, since we apply annealing steps in air during the OPV fabrication and given the sensitivity of Ag to potential oxidation, which can result in the drop of the electrical conductivity, we examined the impact of the 140 °C annealing step on the electrical resistance of the fabricated layers for a timescale longer than the highest temperatures used in our OPV fabrication process. For this, we fabricated a AgNW layer, a first baking step of 50 °C was applied, and the annealing step of 140 °C/90 s in air was followed, giving an initial sheet resistance of 12.0 ohm/sq. Then, we examined the evolution of AgNW layer sheet resistance over time while being exposed at 140 °C. The sample was annealed at 140 °C in air for 5, 10, and 20 min, resulting in average sheet resistances of 11.7, 12.1, and 11.8 ohm/sq, respectively. Given that the average standard deviation of the measurements is ∼0.6 ohm/sq, it is inferred that the effect of 140 °C on electrical resistivity is negligible at this timescale. Also, the transmittance of the film for the various annealing steps remains unaffected in the wavelengths from 300 to 800 nm, as shown in Figure S1. The measurements were performed on uncovered AgNW films that are more prone to oxidation, and thus, we are not expecting a drastic decline in AgNW’s electrical conductivity or optical transmittance during the annealing steps of the other layers in the OPV structure.

To find the optimum AgNWs-based transparent electrodes for use in OPV devices, we selected to examine levels of optical transmittance that ranges between 80 and 87% (similar levels to ITO) and sheet resistance between 7 and 14 ohm/sq. Initially, normally structured OPVs were fabricated using the well-studied P3HT:PCBM active layer material system and various AgNW layer thicknesses (various spin-coating speeds and sequential depositions). For this structure, the AgNWs were effectively covered with thick PEDOT:PSS (150 nm) to avoid possible shunt due to the contact of AgNW with the active layer or the top electrode. Figure S2a illustrates the *J*–*V* characteristics of the representative OPVs incorporating one, two, three, or four times spin-coated AgNWs at 5500 RPM and the reference device incorporating sputtered ITO. From the light and dark *J*–*V* curves (Figure S2b), it can be observed that by increasing the AgNW density (sequential depositions), both the series resistance (*R*_s_) and parallel resistance (*R*_p_) are decreasing. The former is in accordance with the declining sheet resistance of the films for each additional deposition, while the latter can be attributed to higher leakage current as a result of higher probability of peaks present at the AgNW films. Table S2 shows the photovoltaic parameters of the fabricated OPVs. Based on the processing tools and conditions used in our laboratory (please see Experimental Methods), the ITO-free solar cells with two times deposited AgNWs exhibited the highest photovoltaic parameters for the AgNWs-based devices (Table S2). These conditions correspond to 87% transmittance and 12 ohm/sq sheet resistance of the AgNW layer. The best PCE obtained for these ITO-free normal device structure P3HT:PCBM-based solar cells was 2.81%, whereas the reference ITO-based OPV exhibited 3.44%. The corresponding average values over 16 devices are 2.50 and 3.31%, respectively, due to the decline in all PV parameters. The distributions of the respective PV parameters for each normal device structure are presented in Figure S3. The AgNWs-based OPV performance is lower than the reference OPVs since the purpose of this study was to optimize the AgNW contact and not the AgNWs/PEDOT:PSS electrode omitting the optimization of the electrical properties and the thickness of the PEDOT:PSS hole-selective layer.

Inverted organic photovoltaics (OPVs) allow more flexibility on designing the roll-to-roll production process of OPVs, and importantly, OPVs are more environmentally stable, providing technological opportunities compared to normally structured OPVs.^29−31^ In inverted OPVs, metal oxides are used in the cathode as electron-selective contacts in contrast with normally structured OPVs, which use the hydrophilic PEDOT:PSS layer in the anode as the hole-selective contact.^32,33^ Therefore, the same two-time deposited optimized AgNW layer was applied for inverted structured OPVs incorporating ∼40 nm ZnO or ATO/PEI as electron-selective contacts. Almost all the AgNWs-based inverted OPVs with ZnO or thin ATO/PEI were shunted, and hero solar cells provided a PCE of 0.38% (Table S3).

**Figure 2:**
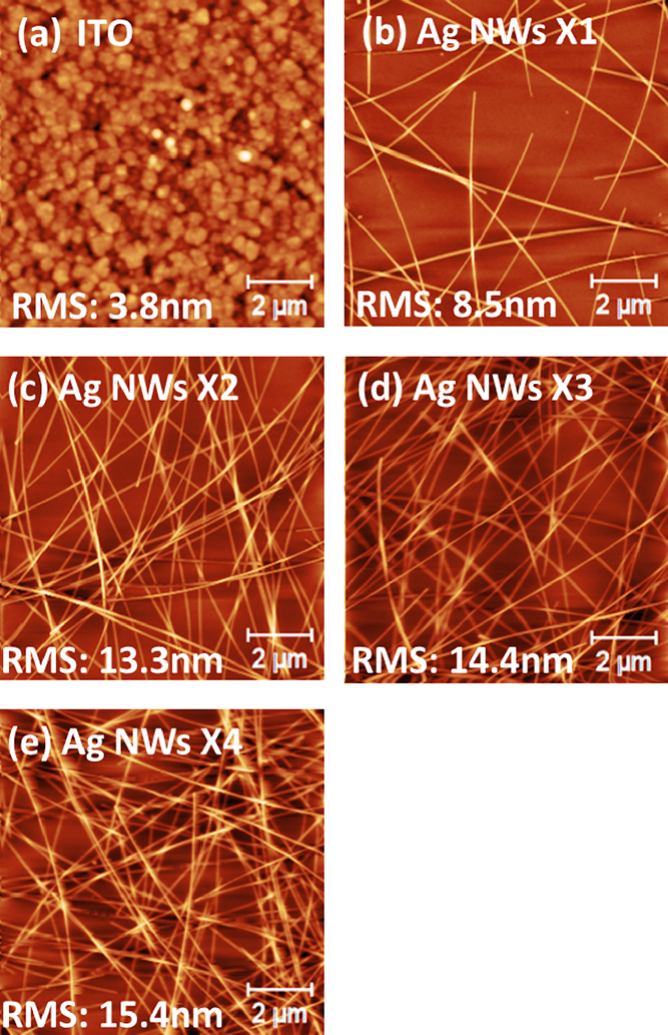
AFM images of (a) ITO and AgNWs deposited (b) one, (c) two, (d) three, and (e) four times.

To gain a better understanding of the fabricated AgNW structure, AFM surface topography images of the AgNWs-based electrodes under investigation were obtained. Figure 2a–d illustrates the images of the sputtered ITO and AgNW films fabricated by applying one, two, three, and four sequential depositions at 5500 RPM, respectively. The density of deposited AgNWs for one spinning is low, in accordance with the highest sheet resistance as shown in Table S1, exhibiting a root mean square (RMS) of 8.5 nm. The RMS of the AgNWs for two, three, and four times coated layers is in the range of ∼15 nm independently of the number of coating steps. In contrast, the surface topography of ITO indicates a much smoother layer with ∼3.8 nm RMS. Furthermore, the profile of ITO and AgNWs coated two times is illustrated in Figure S4a,b, respectively. The AgNW-percolated network consists of a film with areas without AgNW (thin), areas with two AgNWs overlapping (thicker/higher), and parts with multiple AgNWs overlapping (higher). As expected, the morphology of AgNW layers is dominated by spikes that are in the range of 60–70 nm because of the AgNWs packing on the glass surface. It is worth mentioning that the surface energy of the glass substrate affects the morphology, adhesion, and final film formation (sheet resistance and transparency) of the AgNW film independently of the fabrication process of AgNWs (spin-coating speeds and sequential depositions). Therefore, the above studies may vary depending on processing conditions and the choice of the substrate. The above results were obtained using a soda lime glass substrate.

However, spikes and rough surfaces are considered vulnerable part of the device that can result in high leakage current or shunted devices. Such processing issues become more prominent on large-area module processing; thus, for improved solution-processed reliability and solar cell/module device performance reproducibility, metal-oxide buffer layers (150 nm) are preferable for the roll-to-roll production process of OPVs.^25^ For large-scale processes, inverted device structures are preferred, where usually thin (20–40 nm) electron-selective undoped metal oxides such as ZnO are used, rendering the full coverage of the AgNWs challenging. On the other hand, using thicker ZnO electron-selective contact within the inverted ITO-free OPV structure results in lower PCEs due to its low conductivity.^34^ Highly efficient OPVs with ZnO as the electron-selective contact were achieved with the AgNWs-embedded process.^35^ However, the embedding process demands additional processing steps and complicates the low-cost OPV production process. In this paper, we show that the proposed thick ATO/PEI electron-selective contact results in high-performance AgNW inverted ITO-free OPVs using truly simplified and effective solution processing by eliminating the need for embedding the AgNWs.

### 2.2 Silver Nanowires/Antimony-Doped Tin Oxide/Polyethylenimine ITO-Free Electrode for PM6:Y6 Inverted Organic Photovoltaics

As discussed above, the direct use of AgNWs in ITO-free inverted OPVs requires thick solution-based carrier-selective contacts. We examine the impact of a thick (130 nm) ATO/PEI electron-selective contact fabricated on top of the AgNW-based bottom electrode. The doped metal-oxide ATO formulation dissolved in a mixture of butanols was synthesized by Avantama, and an overview of the ink’s properties is presented in Table 1.

**Table 1:** Property Overview of the ATO Metal-Oxide Ink

metal-oxide composition	solvent	grain size (XRD) [nm]	mean hydrodynamic particle size [nm]	viscosity of dispersion [mPa·s]
10 mol % Sb-doped SnO_2_	butanol	6	19	3.2

**Figure 3:**
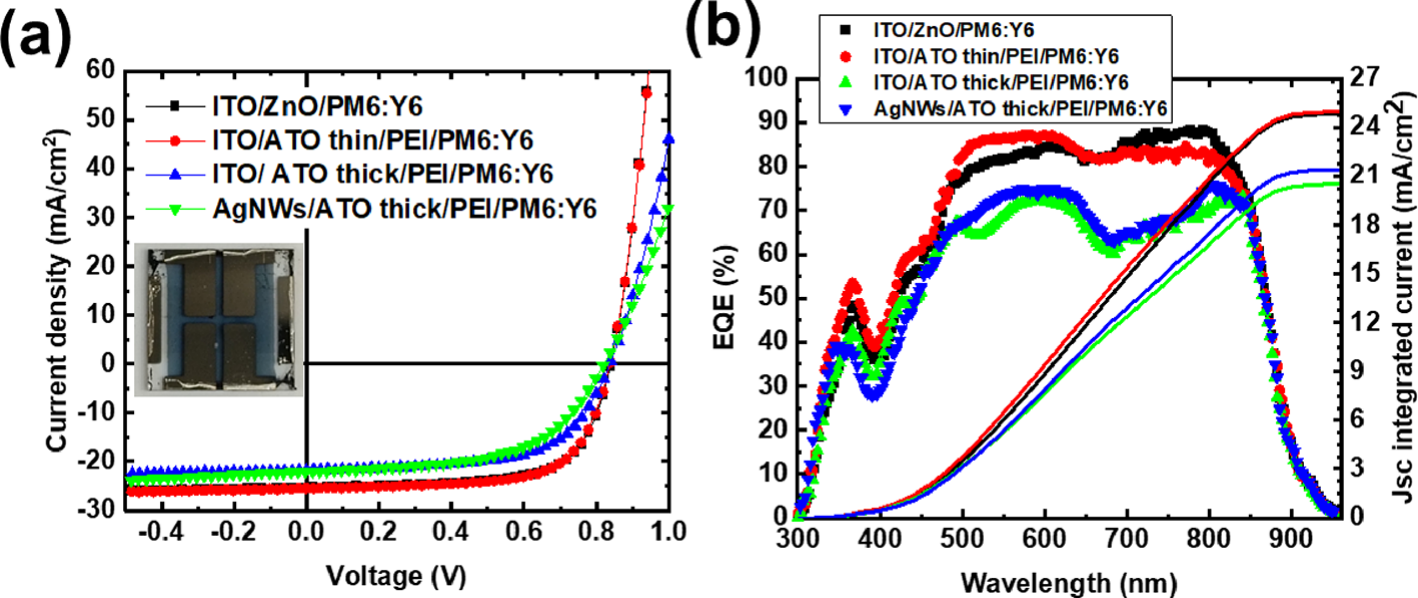
(a) Illuminated *J*–*V* characteristics of hero inverted OPVs with ITO or AgNWs as the bottom transparent conductor and ZnO or ATO/PEI as the electron-selective contact (inset: picture of the device design) and (b) EQE and *J*_sc_ integrated current of the corresponding devices.

After having optimized the AgNW deposition process (described in Section 2.1), a highly efficient non-fullerene acceptor-based PM6:Y6 active layer was introduced in our study. The layer stacking of the inverted OPV devices is AgNW or ITO/ATO/PEI/PM6:Y6/MoO_3_/Ag, while for the reference devices, ZnO was used as the electron-selective contact in the reference inverted OPV structure ITO/ZnO/PM6:Y6/MoO_3_/Ag. For ITO-based inverted OPVs, PCEs similar to the reference ITO/ZnO-based device were achieved by replacing ZnO with a thin (∼40 nm) ATO/PEI layer. As shown in Figure 3a and Table 2, the implementation of a thin ATO/PEI electron-selective layer in inverted ITO-based OPVs delivers the best PCE of 14.44%, similar to the ITO/ZnO-based device that delivers 14.38%. The corresponding average values calculated over 16 devices are 13.54 and 13.53%. However, when a thin ATO/PEI was deposited on top of AgNWs, all the resulted devices were shortened. The issue with these devices is the inability of the thin ATO/PEI layer to fully cover the AgNWs. Thus, using a thicker (130 nm) ATO/PEI layer is a feasible strategy to overcome the abovementioned limitation. The implementation of a thick (130 nm) ATO/PEI electron-selective contact in ITO-based inverted OPVs induces a drop on both best and average PCEs (11.27 and 9.86%, respectively) compared to thin ATO/PEI. This drop is mainly ascribed to higher series resistance induced by the thicker ATO, given that the incident light that reached the active layer is similar due to the very high transparency of the ATO layer even for thick layers.^26^ Next, ITO was replaced by two times deposited AgNW electrode, resulting in 10.23% for the best device as shown in *J*–*V* characteristic curves in Figure 3a with an average over 16 devices of 9.42%. The boxplot of the respective PV parameter distribution for each inverted device structure is presented in Figure S5. The difference between the best PCE values of ITO/thick ATO/PEI- and AgNW/thick ATO/PEI-based devices accounts for a limited reduction of around 9%. The decline is mostly derived from the FF reduction from 63.4 to 58% (Table 2), mainly ascribed to the higher electrical resistance (∼12 ohm/sq) of the two times solution-processed AgNW compared to 4–5 ohm/sq of the ∼250 nm sputtered ITO. Moreover, AFM images of PM6:Y6 for the various underlayers were obtained to examine whether the topography of the active layer changes a parameter, which subsequently could affect the device performance. As shown in Figure S6a,b, the RMS values of PM6:Y6 on ITO/thick ATO/PEI and AgNWs/thick ATO/PEI are similar, implying that the active layer formation on top of the thick ATO/PEI layer is not affected by the type of conductive electrode (ITO or AgNWs coated two times) in the case that a thick buffer layer is applied within the device architecture. This is attributed to the thickness of the ATO layer that can fully cover AgNWs and planarize the surface. In contrast, for the AgNWs/thin ATO/PEI layer, the RMS of the active layer is much higher than that of ITO/thin ATO/PEI due to the inefficient cover of the AgNWs by thin ATO/PEI. This is also indicated by the observation of linear features on the image of AgNWs/thin ATO/PEI that can be attributed to AgNW underlayer structures. The above finding is consistent with the obtained shunted devices when AgNWs/thin ATO/PEI is used as the bottom contact. We note that the two times AgNWs-coated step used in the above reported data is based on the processing tools and conditions used within this paper. According to Cambrios and many of their large-area processing partners, ≤10 ohm/sq is routinely achieved with sheet and roll-to-roll (R2R) AgNW processing with just one coating step (Karl Pichler of Cambrios private communications). The inset picture in Figure 3a depicts the design of the devices, showing the four different solar cells formed on each substrate.

**Table 2:** Photovoltaic Performance Parameters of Inverted OPVs with ITO or AgNWs as the Bottom Transparent Conductor and ZnO or ATO/PEI as the Electron-Selective Contact^b^

OPV	*V*_oc_^a^ (V)	*J*_sc_^a^ (mA·cm^–2^)	FF^a^ (%)	PCE^a^ (%)
ITO/ZnO/PM6:Y6	0.82 (0.81)	25.23 (24.15)	69.5 (69.08)	14.38 (13.54)
ITO/thin ATO/PEI/PM6:Y6	0.82 (0.82)	25.51(24.38)	69.0 (67.78)	14.44 (13.53)
ITO/thick ATO/PEI/PM6:Y6	0.82 (0.79)	21.70 (19.99)	63.4 (62.43)	11.27 (9.86)
AgNWs/thick ATO/PEI/PM6:Y6	0.80 (0.79)	22.05 (20.65)	58.0 (57.59)	10.23 (9.42)

a The data in parentheses are the average values obtained from 16 devices.

b The values in the parentheses are the average over 16 devices.

Figure 3b presents the EQE measurements of the OPV devices under study. The calculated current densities from the EQE measurements are 24.97, 24.82, 20.51, and 21.39 mA/cm^2^ for the devices based on ITO/ZnO, ITO/thin ATO/PEI, ITO/thick ATO/PEI, AgNWs/thick ATO/PEI electron-selective contacts, respectively. The calculated *J*_sc_ current densities for the respective devices are in close agreement with the *J*_sc_ obtained from the *J*–*V* characterization. The ITO/ZnO- and ITO/thin ATO/PEI-based devices produce higher photocurrent compared to ITO/thick ATO/PEI- and AgNWs/thick ATO/PEI-based devices, attributed mainly to lower electrical resistance. Moreover, EQE measurements show a wavelength dependence variation for the different electron-selective contacts. This effect can be ascribed to optical effects such as cavity interference effects and parasitic absorption of the various stacks.^36^

**Figure 4:**
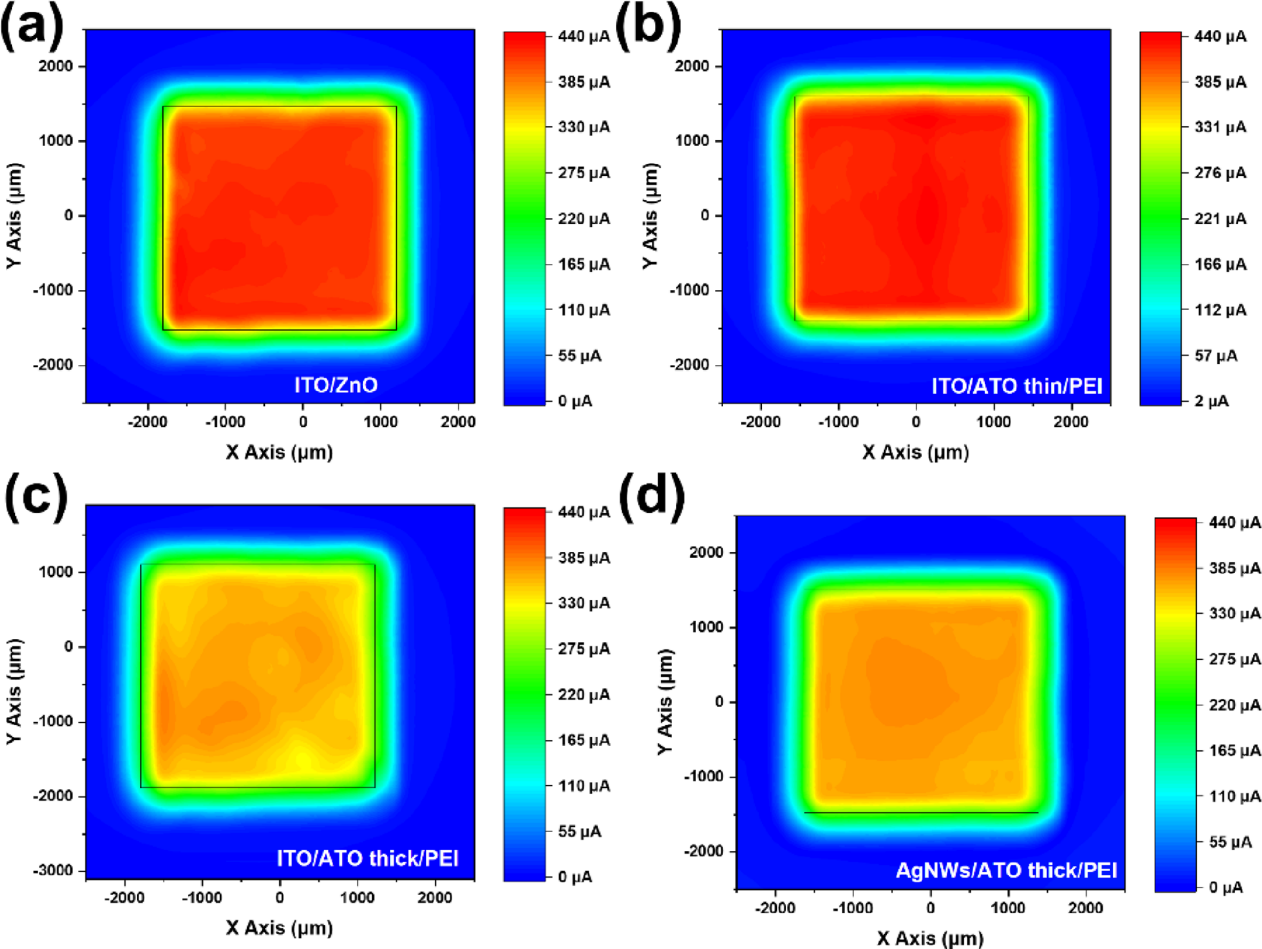
Photocurrent mapping of inverted OPV devices with (a) ITO/ZnO, (b) ITO/ATO/PEI, (c) ITO/thick ATO (130 nm)/PEI, and (d) AgNWs/ATO (130 nm)/PEI as bottom transparent electrodes.

Photocurrent mapping images were obtained to visualize the current distribution of the fabricated OPVs. As presented in Figure 4, the highest photocurrent is obtained for the ITO-based inverted OPVs incorporating thin ZnO and ATO/PEI electron-selective contacts, in agreement with the solar cell device performance data presented in Table 2. The homogeneous current distribution of the well-defined solar cell active region for the inverted ITO-free OPVs using a AgNWs/thick ATO/PEI bottom electrode indicates well dispersed AgNWs that are able to provide continues charge conduction paths and efficient electron carrier selectivity of the proposed ITO-free bottom electrode.

Dong et al. have demonstrated ITO-free inverted OPVs with a PCE of 11.6% for the best performing devices, and the corresponding ITO reference device delivered a PCE of 12.3%. The OPV architecture was AgNWs/ZnO/PM6:IT-4F/MoO_3_/Ag where for the fabrication of AgNWs/ZnO front contact, they applied the embedding method to drastically reduce the roughness of the AgNWs, enabling the deposition of a thin ZnO electron-selective layer. The non-embedded devices showed a low yield of functional devices in agreement with our results.^35^ Yet, the embedding method is challenging for large-scale fabrication, increasing the complexity and restricting the practical production process. The proposed method of non-embedded AgNWs/thick ATO/PEI can be considered as a facile low-temperature solution process, alternative to the typical sputtered ITO/electron-selective contact, providing flexibility and roll-to-roll compatibility for future development of large-area ITO-free inverted OPVs. The PCEs of the best performing devices surpass 10%, with the corresponding ITO/thick ATO devices delivering 11.27%. For the current study, the spin-coating methods were selected for the fabrication of the AgNWs, a non-scalable and material-consuming technique compared to other techniques such as blade coating and slot-die.^37^ Nevertheless, the scope of this study was to investigate the feasibility of combining the AgNW with a thick ATO/PEI layer for efficient inverted OPVs. According to the presented results, it was confirmed that by incorporating a thick ATO/PEI electron-selective contact, it can overcome the difficulty arising from high AgNW roughness. Hence, the fabrication process is simplified by eliminating the need for embedding the AgNW.

## 3 Conclusions

A detailed study of AgNW bottom transparent electrode properties and processing optimization process is presented. A AgNW electrode with 87% transmittance and 12 ohm/sq sheet resistance was found to be suitable for ITO-free OPV implementation. Furthermore, the inverted OPV AgNWs ITO-free bottom electrode requirements to use a thicker and higher conductive carrier-transporting layer than commonly used metal oxides and avoid the AgNW-embedded process were fulfilled by incorporating a thick ATO/PEI electron-selective contact. The proposed fully solution-processed bottom transparent electrode for inverted OPVs consists of non-embedded AgNWs and ATO/PEI as the electron selective contact. Finally, the developed non-embedded AgNWs/ATO/PEI ITO-free bottom electrode with simplified processing and up-scalability perspectives was combined with the NFA PM6:Y6 active layer material system, resulting in ITO-free inverted OPV exhibiting a PCE above 10%.

## 4 Experimental Methods

### 4.1 Materials

Prepatterned glass-ITO substrates (sheet resistance, 4–5 ohm/sq) were purchased from Psiotec Ltd. NKA710 Silver Nanowires ink was purchased from Cambrios Film Solutions, P3HT from Rieke Metals, PBDB-T-2F (PM6) from Ossila Ltd., BTP-4F (Y6) from Solarmer, and PC[60]BM from Solenne BV. PEI and all the other chemicals used in this study were purchased from Sigma-Aldrich. Antimony-doped tin oxide (10 at % Sb:SnO_2_) solution in a mixture of butanols was developed by Avantama (ATO, product no. 10095). More details for the synthesis, properties, and characterization of ATO can be found elsewhere.^25,26^

### 4.2 ZnO Sol–Gel Synthesis

The ZnO films were prepared using zinc acetate dehydrate and monoethanolamine as a stabilizer dissolved in 2-methoxyethanol. Zinc acetate dehydrate (0.05 g) was mixed with 0.0142 g of monoethanolamine and dissolved in 0.5 mL of 2-methoxyethanol. The resulting precursor solution was stirred at room temperature for 20 min in ambient conditions, then coated, using doctor blading, on top of ITO substrates, and annealed after deposition at 140 °C for 20 min in air, forming a 40 nm-thick ZnO layer.

### 4.3 Solar Cell Processing

The inverted OPVs under study were ITO or AgNWs/ETL/active layer/MoO_3_/Ag. ITO and glass substrates were sonicated in acetone and subsequently in isopropanol for 10 min. AgNWs were spin-coated on soda lime glass substrates. The films were dried for 90 s at 50 °C and annealed for 90 s at 140 °C. The ATO solution was deposited by doctor blading at 70 °C and annealed for 20 min at 120 °C. The PEI interfacial layer was spin-coated, resulting in an ultrathin interfacial layer, and annealed at 100 °C for 10 min.^26^ For the normal devices, PEDOT:PSS was doctor-bladed and then annealed at 140 °C on a hotplate for 20 min in ambient conditions. The thickness of PEDOT:PSS was ∼50 nm for the ITO-based devices and 150 nm for the AgNWs-based OPVs. The active layer solution of conventional P3HT:PCBM (1:0.8) with 27 mg/mL concentration was deposited on the ETL by doctor blading, resulting in a film with a thickness of ∼200 nm, and annealed for 20 min at 140 °C. For the PM6:Y6 inverted OPV devices, the AgNW electrodes were fabricated by two times spin-coating at 5500 RPM and dried at 50 °C for 90 s followed by annealing at 140 °C for 90 s in air. The active layer PM6:Y6 (1:1.2) was spin-coated from a 16 mg/mL solution in chloroform, resulting in a film thickness of ∼110 nm, and annealed for 10 min at 100 °C. Finally, 10 nm MoO_3_ and 100 nm Ag layers were thermally evaporated through a shadow mask to finalize the devices, giving an active area of 0.9 mm^2^. Only normally structured OPVs were encapsulated. Encapsulation was applied directly after evaporation in the glove box using a glass coverslip and an Ossila E131 encapsulation epoxy resin activated by 365 nm UV irradiation.

### 4.4 Characterization

The thickness of the films was measured with a Veeco Dektak 150 profilometer. AFM images were obtained using a Nanosurf Easyscan 2 controller under tapping mode. Electrical conductivity measurements were performed using a four-point microposition probe, Jandel model RM3000. For illuminated current density–voltage (*J*–*V*) characteristics, a calibrated Newport Solar simulator equipped with a Xe lamp was used, providing an AM1.5G spectrum at 100 mW cm^–2^ as measured by a certified Oriel 91150V calibration cell. Transmittance measurements were performed with a Shimadzu UV-2700 UV–Vis optical spectrophotometer. EQE measurements were performed by Newport System, Model 70356_70316NS. Photocurrent mapping measurements were performed under 405 nm laser excitation wavelength, 50% laser intensity, and 40 μm laser spot size using a Botest PCT photocurrent system.

## Supporting Information Available

Table S1: sheet resistances of the ITO and one, two, three, and four times spin-coated AgNWs; Figure S1: transmittance of AgNWs on glass of the fresh samples and after 6, 10, and 20 min of annealing at 140 °C in air; Figure S2: light and dark *J*–*V* curves obtained from the OPVs with ITO and one, two, three, and four times spin-coated AgNWs in normal device structure AgNWs/PEDOT:PSS/P3HT:PCBM/Ca/Al; Table S2: corresponding solar cell parameters; Figure S3: boxplots of the PV parameters *V*_oc_, *J*_sc_, FF, and PCE for the normal structure devices incorporating ITO (reference) and AgNW deposited one, two, three, and four times as the front contact electrode; Table S3: light *J*–*V* solar cell parameters of the hero ITO-free inverted device AgNWs/thin ATO:PEI/P3HT:PCBM/Ca/Al; Figure S4: AFM image profiles of ITO and AgNWs deposited two times; Figure S5: boxplots of the PV parameters *V*_oc_, *J*_sc_, FF, and PCE for the inverted structure devices incorporating ITO/ZnO (reference), ITO/thin ATO/PEI, ITO/thick ATO/PEI, and AgNW/thick ATO/PEI as the front contact electrode; Figure S6: AFM image profiles and corresponding RMS of PM6:Y6 deposited on top of ITO/thick ATO, AgNW/thick ATO, ITO/thin ATO, and AgNWs/thin ATO (PDF)
