# Supplementary material for: Non-Embedded Silver Nanowires/Antimony-Doped Tin Oxide/Polyethylenimine Transparent Electrode for Non-Fullerene Acceptor ITO-Free Inverted Organic Photovoltaics
Source: ACS Appl Electron Mater. 2023 Jan 11;5(1):181–8. doi: 10.1021/acsaelm.2c01187 (PMC9878715; doi:10.1021/acsaelm.2c01187)
Supplement: Supplementary file 1 — el2c01187_si_001.pdf [file el2c01187_si_001.pdf]

Supporting Information

Non-Embedded Silver Nanowires/Antimony doped Tin Oxide/Polyethylenimine Transparent Electrode for Non-Fullerene Acceptor ITO-free Inverted Organic Photovoltaics

[1] *Efthymios Georgiou [1], Apostolos Ioakeimidis [1], Ioanna Antoniou [1], Ioannis T. Papadas [1,2], Alina Hauser[3], Michael Rossier[3], Flavio Linardi[3], and Stelios. A. Choulis[1,]**

[1] Molecular Electronics and Photonics Research Unit, Department of Mechanical Engineering and Materials Science and Engineering, Cyprus University of Technology, 45 Kitiou Kyprianou Street, Limassol, 3603, Cyprus

[2] Department of Public and Community Health, School of Public Health, University of West Attica, Athens, Greece

[3]Avantama AG, Laubisruetistr. 50, Staefa 8712, Switzerland

* Corresponding Author E-Mail: stelios.choulis@cut.ac.cy

Table S1: Sheet Resistance values of AgNWs films, x1, x2, x3 and x4 and commercial ITO.

AgNWs	x1	x2	x3	x4	ITO
**Sheet Resistance (Ohms/Sq)**	28	12	8.5	7	4 -5

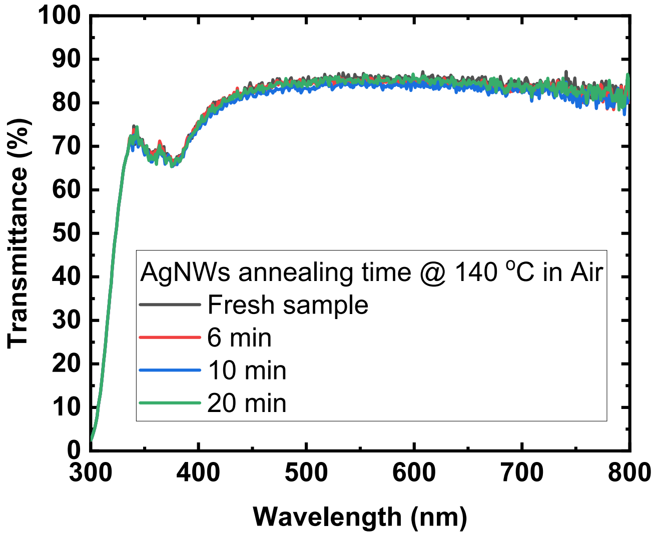

Figure S1 Transmittance of AgNWs on glass of the fresh samples and after 6, 10 and 20 min of annealing at 140 °C in air.

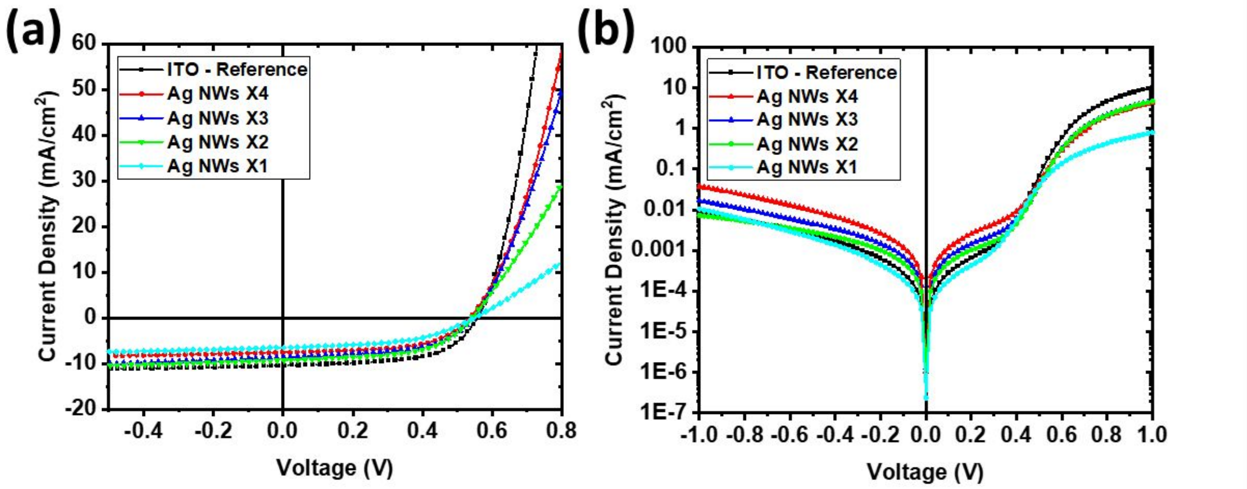

Figure S2 (a) illuminated and (b) dark JV characteristics of normal structured OPVs with Ag NWs deposited x1, x2, x3, and x4 times. Device structure: ITO or Ag NWs /PEDOT:PSS/P3HT:PCBM/Ca/Al

Table S2: Photovoltaic parameters of normal structured OPVs with Ag NWs deposited x1, x2, x3 and x4 times. Device structure: ITO or Ag NWs/PEDOT:PSS/P3HT:PCBM/Ca/Al. The values in the parentheses are the average over 16 functional devices.

Normal OPVs	Voc (V)	Jsc (mA.cm-2)	FF (%)	PCE (%)
**ITO – Reference**	0.56 (0.57)	10.37 (9.70)	58.9 (60.26)	3.44 (3.31)
**Ag NWs – 4x**	0.54 (0.55)	7.54 (6.86)	56.3 (54.17)	2.31 (2.03)
**Ag NWs – 3x**	0.54 (0.55)	8.63 (7.36)	54.6 (55.41)	2.57 (2.23)
**Ag NWs – 2x**	0.54 (0.56)	9.34 (7.91)	55.3 (56.40)	2.81 (2.50)
**Ag NWs – 1x**	0.54 (0.53)	6.56 (6.51)	50 (43.15)	1.78 (1.49)

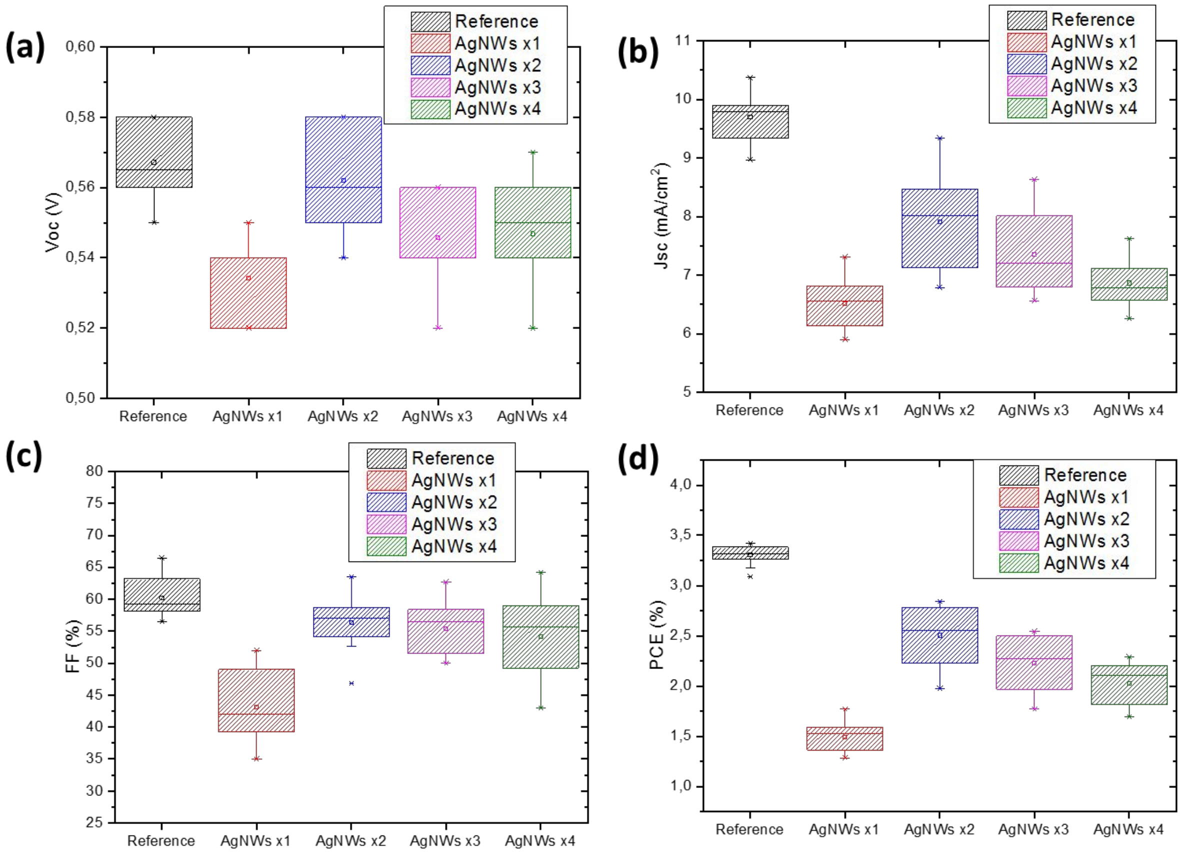

Figure S3 Box-plots of the PV parameters for normal device architecture P3HT:PCBM based solar cells (a) Voc, (b) Jsc, (c) FF and (d) PCE for the P3HT:PCBM normal structure devices incorporating ITO (reference), AgNW x1, x2, x3, x4 as front contact electrode.

Table S3: Photovoltaic parameters of hero inverted conventional P3HT:PCBM, ITO-free OPVs with Ag NWs deposited x2 and thin ATO:PEI electron selective contact (Device structure: Ag NWs/thin ATO:PEI/P3HT:PCBM/Ca/Al). As indicated also within the main text almost all Ag NWs/thin ATO:PEI based inverted ITO-free OPVS were shunted. While the hero ITO-free OPVs provided very limited PCE as represented in the table below.

**Hero Inverted OPVs**	**Voc (V)**	**Jsc (mA.cm^{-2})**	**FF (%)**	**PCE (%)**
AgNWs/thin ATO(40 nm) /PEI	0.26	3.44	42.2	0.38

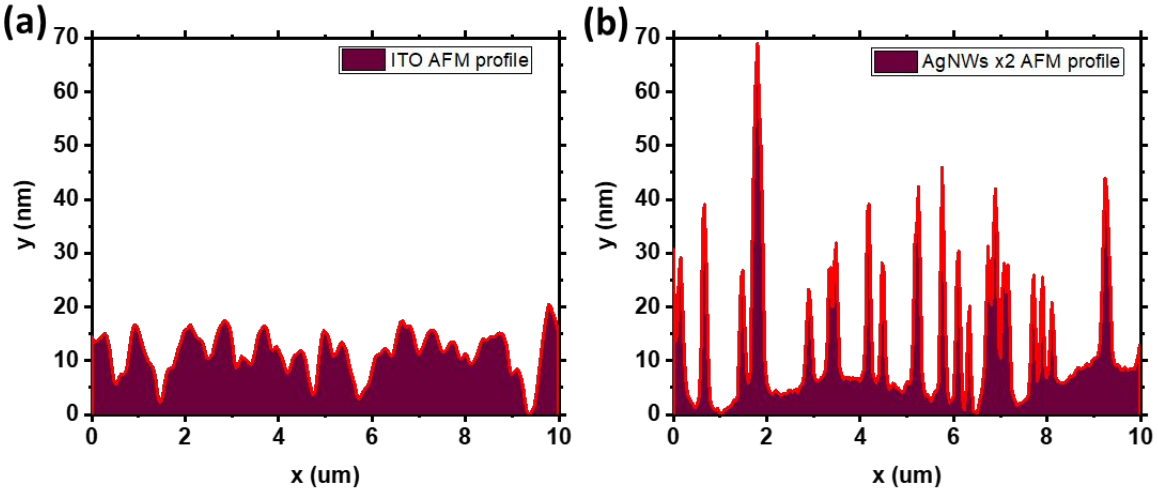

Figure S4 AFM image profiles of (a) ITO and (b) Ag NWs deposited X2

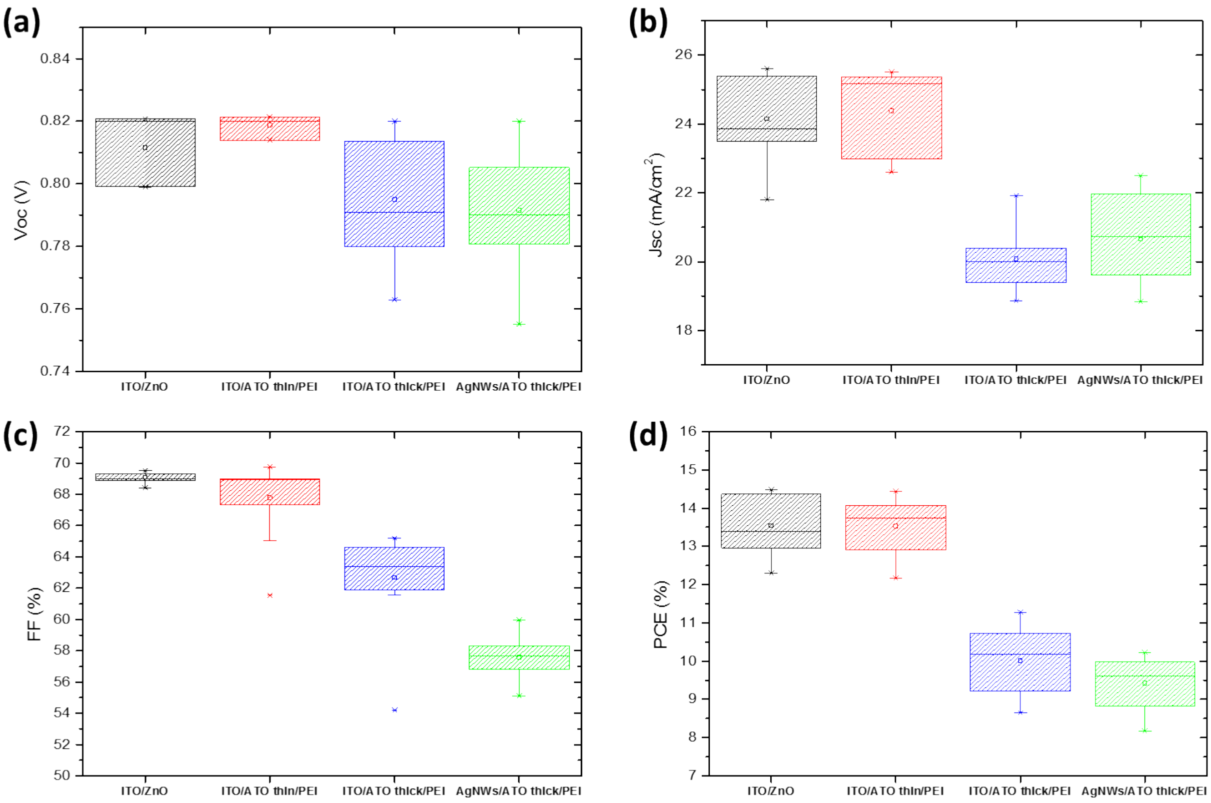

Figure S5 Box-plots of the PV parameters for inverted device architecture PM6:Y6 based solar cells (a) Voc, (b) Jsc, (c) FF and (d) PCE for the inverted structure PM6:Y6 devices incorporating ITO/ZnO (reference), ITO/thin ATO/PEI, ITO/thick ATO/PEI and AgNW /thick ATO/ PEI as front contact electrode.

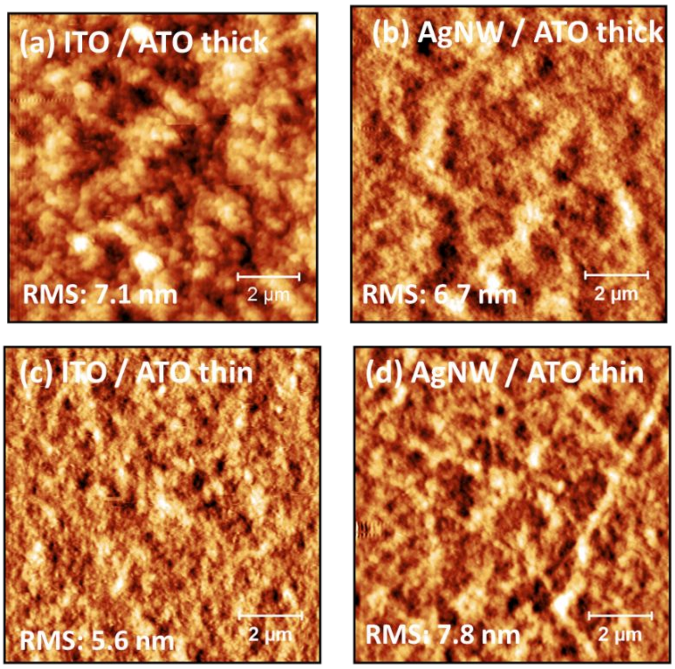

Figure S6 AFM image profiles and the corresponding RMS of PM6:Y6 deposited on top of (a) ITO/ATO thick, (b) AgNW/ATO thick, (c) ITO/ATO thin and (d) AgNWs/ ATO thin
